# Large Bias in Matching Small Horizontal and Vertical Extents Separated in Depth in the Real World Is Similar for Upright and Supine Observers

**DOI:** 10.3390/vision9010011

**Published:** 2025-02-03

**Authors:** Frank H. Durgin, Chung Sze Kwok, Katelyn M. Becker, Ya Min Phyu

**Affiliations:** Department of Psychology, Swarthmore College, 500 College Ave., Swarthmore, PA 19081, USA; ckwok1@swarthmore.edu (C.S.K.); kbecker2@swarthmore.edu (K.M.B.); yphyu1@swarthmore.edu (Y.M.P.)

**Keywords:** horizontal–vertical illusion, supine, angular expansion

## Abstract

The apparent sizes of horizontal and vertical lines show an anisotropy known as the horizontal vertical illusion (HVI) wherein vertical lines appear to be longer than their horizontal counterparts. Whereas a typical HVI comparing vertical and horizontal lines in a plane produces a 5–10% illusion, a much larger-scale illusion (15–25%) is often found for large objects in the real world, and this has been related to differential angular exaggerations in perceived elevation (vertical) and azimuthal (horizontal) direction. Recently supine observers in virtual environments were found to show larger exaggerations in perceived azimuth than upright observers. Here, 48 participants were tested in both supine and upright postures in an outdoor environment while matching fairly small physical extents in the real world. They adjusted the magnitude of the horizontal extent to perceptually match fairly small vertical poles (0.7–1.3 m tall) that were either presented at the same viewing distance as the matching extent or in a different depth plane, so that size at a distance had to be compared. Supine observers viewed the scene, as though upright, through a large mirror mounted overhead at 45° that was adjusted to approximate their normal eye height. When the matcher extent was at a different distance than the pole, horizontal extent matches typically exceeded the actual pole height by about 15% or more, whether the viewer was upright or supine. The average overestimation was only about 10% when the matching extent was at the same distance. Despite the similarity in performance across different postures for spatial matching, supine observers gave much higher explicit estimates of azimuthal direction than upright observers. However, although the observation of exaggeration in perceived azimuth for supine observers was replicated in a second study with 24 additional participants using a mirror with a smaller (more normal) aspect ratio, the magnitude of the exaggeration seemed to be greatly reduced when the field of view of the apparatus had a more typical aspect ratio. This suggests that the unusually large exaggeration of azimuth found in a previous report with supine observers may have been caused by the unusually large aspect ratio of the viewing apparatus used.

## 1. Introduction

The apparent sizes of horizontal and vertical lines show an anisotropy known as the horizontal vertical illusion (HVI) wherein vertical lines appear to be longer than their horizontal counterparts [1,2,3,4,5]. Depending on the configuration (e.g., T-shape vs. L-shape) and whether they are attached or separated, the magnitude of this illusion for 2D lines can range from about 1.03 to about a 1.1 bias [6,7,8,9]. These biases are thought to be retinotopically coded [10,11], and there is evidence that they may reflect image statistics consistent with foreshortening of apparently vertical projections of extents in depth along the ground [12,13,14,15].

However, in contrast to the classical illusion that has been studied with lines, when large objects or extents are compared in the real world, much larger perceptual anisotropies (1.15–1.25) are observed [16,17,18,19,20]. In contrast to the smaller illusion with lines, it has been shown that the major portion of this large-scale HVI remains locked to the gravitational environment, even when observers view the objects while lying sideways at their normal eye height [21]. Thus, it appears that the large-scale illusion has a different source.

Importantly, the large-scale HVI (with a gain of up to 1.25) seems to correspond to the differential angular biases in azimuth (1.20) and elevation (1.5) that have been reported in the literature [22,23,24,25]. Because angular variables such as angular declination (the egocentric angular direction below straight ahead where an object makes contact with the ground) is a strong cue for visual distance [26,27,28,29], the observed angular expansion in elevation (with a gain of about 1.5 [22]) can quantitatively explain the underestimation of egocentric ground distance that is normally observed [30,31,32]. Although the large-scale HVI does not obviously seem to depend on perceived ground distance, there is some evidence that it actually does. Specifically, it has been shown in virtual reality that even fairly small (1.5 m) poles will show the large-scale HVI if the comparison pole is presented at a different depth along the ground [33]. That is, it appears that when one cannot simply compare the retinal sizes but must compare size at a distance [34], then the large-scale HVI is found. Apparently, however, for much larger objects, the size-at-a-distance strategy is the default even when no depth separation occurs [20,33].

Because the observation that separation in depth helps to induce the large-scale illusion has only been tested in a virtual environment, the present investigation sought to replicate it in the real world with real objects. That is, a primary goal of this article is to confirm that this finding occurs in the real world.

**Figure 1:**
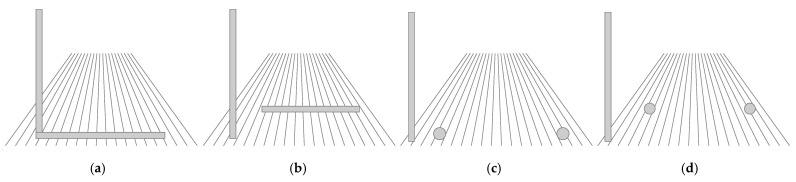
Schematic depiction of 4 configurations with a vertical pole with a horizontal matcher extent (**a**) adjoined in an L-configuration or (**b**) separated by depth [33]. The two right panels show the two configurations tested in the present study by using a separation between two balls that are either (**c**) at the same depth as the vertical pole or (**d**) at a different depth. The perspective lines representing the ground are included here only to help convey the depth relationships of the objects, which were, in all cases, viewed on a (simulated or real) mown grass field.

Two additional goals, however, were incorporated into the main experiment. First, we wanted to test whether the effect depended on separation in depth or whether it could also be produced simply by any separation of the horizontal and vertical objects, given that connectedness alone can reduce the HVI [6]. For this reason, we used horizontal matcher extents that were always separated from the target horizontally but were additionally separated in depth in the depth-separation conditions, as shown schematically in Figure 1, by a vertical displacement representing depth along the ground. It should be noted that it has been shown that a gap works as well as an extent [33].

A further goal of the present study was to test whether supine observers (participants lying on their backs) would show a different pattern of HVI than those tested in an upright posture. One previous study has argued that prone observers (lying on their stomachs with their heads tilted up) showed little or no large-scale HVI [18], though the participants in that study were tested at an abnormally low eye height of about 0.4, rather than 1.4 m. Moreover, a recent study has found that explicit judgments of azimuthal direction (i.e., angular direction to the left or right of straight ahead) appear to be more exaggerated for supine observers than for upright observers [35]. That study did not test the HVI directly, but it did find similar levels of exaggeration of perceived azimuth and of perceived elevation (i.e., with a gain of about 1.5) for supine observers in a virtual environment. We therefore chose to test supine observers in the real world as well, using a cart apparatus to hold them near normal eye height and a mirror system so that they could see the scene as though standing upright.

## 2. Materials and Methods

### 2.1. Preregistration and Open Science

Two experiments are described here, using a similar apparatus and visual scene. Both experiments were preregistered on aspredicted.org (https://aspredicted.org/zcs5-3fwp.pdf and https://aspredicted.org/cnwz-gzdm.pdf, accessed on 28 January 2025). Full data and analysis files are available on OSF (https://osf.io/zgjv3/?view_only=2d1bf1b2e9f947cca84c1a0fe4fe20cd, accessed on 28 January 2025). The study was approved by the Swarthmore College IRB under protocol IRB-FY22-23-14. All data were collected in the summer of 2024.

### 2.2. Participants

#### 2.2.1. Sample Sizes

Although the primary preregistered analysis was Bayesian, a sample size of 48 was chosen for the main experiment based on a design requiring a multiple of 8 for balance, and a power analysis using GPower 3.1 [36] with 80% power and a chance of obtaining an effect with an estimated effect size of 0.6 for half the sample of participants, to test the replication of the effect of depth separation, based on data from the prior study [33]. For the follow-up experiment examining perceived azimuth, a sample size of 24 was chosen. All participants wore corrective lenses as needed. There were no other restrictions.

#### 2.2.2. Participants in Experiment 1

Forty-eight Swarthmore College students, aged 19–22 years (M = 20.2 years), participated in the experiment in exchange for payment. Of the 48 participants, 22 (45.8%) identified as White, 17 (35%) as Asian or Asian American, 9 (19%) as Hispanic, Latino, or Spanish origin, 9 (19%) as Black, African, or African American, 1 (2%) as American Indian or Alaska Native, and 1 (2%) did not identify with any of the listed backgrounds. Additionally, 17 (35%) identified as male, 26 (54%) identified as female, 4 (8%) identified as non-binary, and 3 (6%) identified as genderqueer. When asked to indicate their home social class, 16 (33%) identified as low income, 3 (6%) as working class, 11 (23%) as middle class, 13 (27%) as upper middle class, and 5 (10%) as upper class.

#### 2.2.3. Participants in Experiment 2

Twenty-four additional Swarthmore College students, 18–22 years of age (M = 19.5), were paid to participate in the experiment; none had participated in Experiment 1. Of the 24 participants, 11 (46%) identified as White, 13 (54%) as Asian or Asian American, 5 (21%) as Hispanic, Latino, or Spanish origin, and 1 (4%) as Black, African, or African American. Additionally, 9 (38%) identified as men (37.5%), 11 (46%) identified as women, 4 (17%) identified as non-binary, and 2 (8%) identified as genderqueer. When asked to indicate their home social class, 1 identified as low income (4%), 3 (13%) as working class, 7 (29%) as middle class, 11 (46%) as upper middle class, and 2 (8%) as upper class.

### 2.3. Apparatus and Field

Both experiments were conducted in a small grassy field that extended about 12 m from a building behind the participants and terminated at a tall meadow. All participants were tested in both a standing posture and a supine posture.

**Figure 2:**
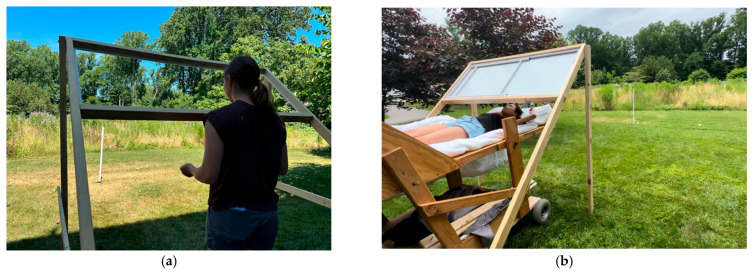
The two viewing conditions for Experiment 1 are shown. On the left (**a**), an experimenter demonstrates the standing condition, viewing the scene through the empty frame. In this example, the matching extent is at a farther distance than the pole. On the right (**b**), another experimenter demonstrates the supine viewing condition with the rectangular mirror mounted in the frame. In this case, the matching extent is at the same distance as the pole. In both cases, the viewer moved the right-hand ball using a fishing line that allowed the ball to be moved either closer or farther from the reference ball. It should be noted that each participant was tested in both postures, and the same depth difference (or absence thereof) between balls and poles was used for both postures for a given participant. Two different pole distances and ball distances were used (5 or 6 m) for all participants.

In the supine posture, participants lay on an elevated cart (see Figure 2) so that they could clearly see the scene in a mirror tilted at 45° overhead. The mirror was 60 × 162 cm in Experiment 1. A round mirror 1.02 m in diameter was used in Experiment 2. The viewing position for supine observers optically approximated that of their standing position (which was measured using a tape measure after they had consented). The positioning of the cart and mirror apparatuses was maintained from participant to participant and day to day by the use of markers (golf tees) embedded in the ground that indicated where the mirror structure and where the cart should be positioned or (for the standing posture) where the participant should stand.

In Experiment 1, the mirror and cart were removed for the standing posture so that participants looked through the empty frame of the mirror at the scene so as to frame the scene equivalently in both postures (see Figure 2). The height of the mirror device was adjustable to accommodate differences in eye height of up to 0.3 m, which was sufficient for our pool of participants.

In Experiment 2, the mirror and cart were placed on one side of the field, and the standing position was on the other side, with all angle judgments being made toward the main part of the field. These positions were swapped from left to right for half the participants.

#### 2.3.1. Materials for the HVI Task (Experiment 1)

White PVC pipe was used to create 6 vertical stimuli with heights of 0.7, 1.0, and 1.3 m and diameters of 2.5 or 3 cm. A chemistry stand (hidden inside the pipe) was used to hold the stimuli upright from the ground. One stimulus was presented at a time. Horizontal extents were defined by the distance between two white styrofoam balls (6 cm diameter). A fixed ball was presented 15 cm to the right of the pole (either at the same depth or a different depth), and a second, moveable ball was rigged with a fishing line so that participants could easily adjust its position.

All starting ball locations and near and far pole locations were pre-marked with golf tees so they could be easily positioned between trials. Between trials, participants wore a disposable sleep mask so that they did not observe the measurement of their response nor the repositioning of the pole and balls in preparation for the next trial. The experimental scene is shown in Figure 2.

#### 2.3.2. Materials for the Azimuth Estimation Task (Experiments 1 and 2)

The two tallest PVC pipes, capped with large styrofoam balls (12 cm in diameter) were used to represent angular extents. One was presented directly in front of the participant, with the other positioned off to one side.

The same field was used as before, but this time, two different viewing positions were used, with the cart/mirror set up in the left viewing position for half the participants and in the right viewing position for the other half because this allowed for larger angles without having to move the mirror apparatus while testing the participants. The azimuthal angles to be judged from the right viewing position were 8°, 24°, and 40°. The azimuthal angles to be judged from the left viewing position were 16°, 32°, and 48°. The only response given was a verbal (numeric) estimate of the angle between the straight-ahead poles and the test pole in degrees.

**Figure 3:**
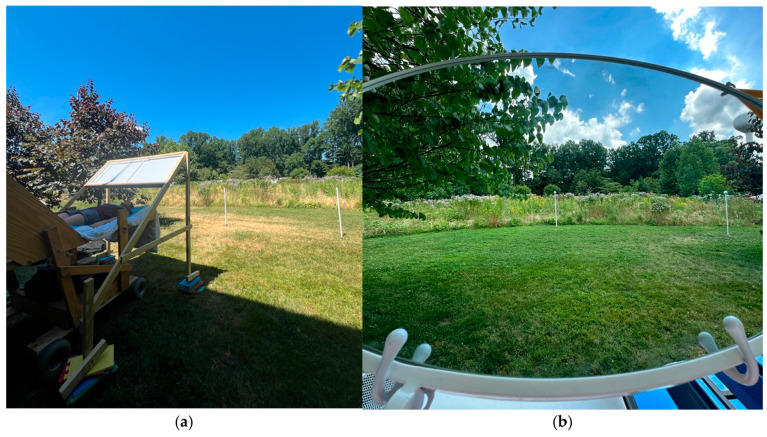
(**a**) The setup for the final angle judgment trial in Experiment 1 (supine posture). The blocks used to raise the mirror to match the participant’s eye height should be noted. The two 1.3 m poles were used to mark the straight ahead and the angular deviation to be estimated verbally. (**b**) The view (for a supine observer) through the round mirror for an azimuth judgment (40°) in Experiment 2.

It should be noted that when the mirror was in the right-side position (as shown in Figure 3b), the extents were to the left when facing the field but appeared to participants to be to the right. Thus, all participants with the mirror in this position made standing judgments to the right and supine judgments that also appeared to them to be to the right (in a mirror-reversed version of the field). Similarly, when the mirror was on the left, all judgments whether standing (on the right) or supine (on the left) appeared to the participants to be to the left of the central target from both viewing perspectives.

### 2.4. Experimental Design (Experiment 1)

Each participant in Experiment 1 was assigned by alternation to one of four conditions involving both (1) the order in which they completed the two postures and (2) whether they were in the depth-separated condition or the depth-aligned condition. Participants made matches to all six poles (three heights, twice) in random order in one posture and then all six poles in the other posture without immediate repetition of any height. Nuisance variables (diameter of pole, distance to pole, and initial separation of the two matcher balls) were carefully balanced. For example, to control for anchoring effects, in each posture, each pole height was tested once with an extent that was initially half the height of the pole and once with an extent that was initially twice the height of the pole. The observed anchoring effect across all participants and conditions was quite small (~7% median difference based on initial positions differing by a factor of three). Additionally, each pole height for each posture was tested both at a 5 m viewing distance and at a 6 m viewing distance (with the matching-extent balls either at the same distance or at the alternative distance, between participants).

In addition to the HVI experiment, a single trial of perceived azimuthal angle was conducted in whatever posture the participant was in at the end of the experiment. The precise angle tested took the exact viewing position into account but approximated 30°, and the verbal estimate was converted to a ratio to the actual angle for purposes of analysis. This task is illustrated in Figure 3a.

### 2.5. Procedure (Experiment 1)

Upon arriving at the experimental site, participants received verbal instructions before beginning the task and gave signed consent. Their eye height was measured in cm using a tape measure so that the cart and frame could be adjusted. They were instructed to adjust the position of a ball until the gap between it and another stationary ball matched the height of the pole. In delivering the instructions, the experimenters emphasized that it was a perception task, and participants should respond with their best match based on their perception. Before beginning the experiment, participants were allowed to practice using the fishing line to move the ball. Before and after each trial, the participant was instructed to cover their eyes with the sleep mask. After completing the setup for each trial, one experimenter notified the subject that their sleep mask could be removed, handing them the fishing line. Participants were told to take as much time as they needed to make the adjustment and to notify the investigator when they were satisfied, at which point they would hand the fishing line back to the investigator to prevent any accidental movements of the adjustable ball during measurement. They then donned the sleep mask. The gap between the balls was measured in cm, and the distance was recorded. Then, the stimuli for the next trial were set up, and the process was repeated. When it was time to change postures, the participant was led through the process of either dismounting from the cart or mounting the cart after the measurement was complete. The sleep mask was then used while the next trial was being set up by the experimenters.

At the conclusion of the HVI experiment, a single angle estimation task was conducted in whichever posture the subject was currently in (half were supine; half were upright). The stimuli for this were the two tall poles, with the second pole (on a second chemistry stand) situated 3.82 m to the right of the main pole position in the same orthogonal plane (at either 5 or 6 m). Participants were asked to estimate how far the pole was to the right (or left) of the center pole in degrees (it should be noted that the perceived left–right direction of the scene was reversed for those in the supine conditions because of the mirror).

Participants also filled out a demographic questionnaire at the end of the experiment and were paid. The entire procedure took about 20–25 min.

### 2.6. Experimental Design (Experiment 2)

Each participant in Experiment 2 was assigned, by alternating subject numbers, to first make judgments while standing or to first make judgments while supine. The mirror position was switched (from one side of the field to the other) after the first 6 participants, after the next 8 participants, and again after the next 6 participants so that 12 participants were tested with each arrangement, half of whom started in each posture.

The order of the three angles tested from each position was entirely random apart from the fact that three of the angles were always judged from the right side and the other three from the left side (though all six were either left or right from the participant’s point of view, because, when the mirror was to the left side of the field, angles in the mirror appeared to be to the left, rather than to the right, from the participant’s point of view).

The preregistered dependent variable was the slope of the fit line for the three angle judgments made in each posture. After checking for order effects and range effects, the planned analysis was a linear-mixed-effects regression with posture as a within-subject variable and angular range (8 to 40° vs. 16 to 48°) included as a predictor.

### 2.7. Procedure (Experiment 2)

Upon arrival at the study site, participants were given verbal instructions before giving consent and then beginning the task. To help illustrate the task, a bird’s-eye-view diagram was used to indicate the angle to be judged. The diagram always depicted the correct (subjective) direction (left or right) for the participant. It should also be noted that participants were told that the diagram depicted merely an example angle and that the angles they would be asked about would simply be in the direction shown (right or left). Between trials, participants wore a sleep mask so they did not see each angle being set up. After completing three angles in their first position/posture, they were led to the other position/posture to complete the other three angles as before. The entire procedure took about 12 min. Following the experimental trials, participants completed demographic questionnaires and were paid.

## 3. Results

### 3.1. Preregistered Analyses of HVI Data from Experiment 1

The preregistered analysis plan called for two initial Bayesian tests [37,38] seeking to determine the odds that upright participants made horizontal matches that were not less than 1.1 greater than the vertical pole in the depth-separated condition and in the depth-aligned condition. All analyses were carried out in log space unless indicated otherwise. Bayesian tests were selected to make signed hypotheses about the anticipated values to determine the relative odds of the null hypotheses and experimental hypotheses.

The geometric mean match ratio for each participant was computed for their upright posture trials. In the depth-separated condition, the mean ratio was 1.16, and the odds against the null hypothesis (that the HVI in this condition is 1.1 or less) were 64 to 1. This replicates, in the real world, the discovery made using VR that a large-scale HVI can be produced with fairly small objects simply by separating them in depth [33].

In contrast, for participants in the depth-aligned condition, with a geometric mean ratio of 1.09, the odds against the null hypothesis (of an effect no more than 1.1) were only 1.4 to 1. It should be noted that the magnitude of the depth-aligned HVI is similar to that observed for the 2D HVI using spatially separated horizontal and vertical lines with a horizontal depth gradient in the background [4]. It is also similar to the effect observed for similarly sized objects in VR when the horizontal and vertical extents were at the same depth [33].

**Figure 4:**
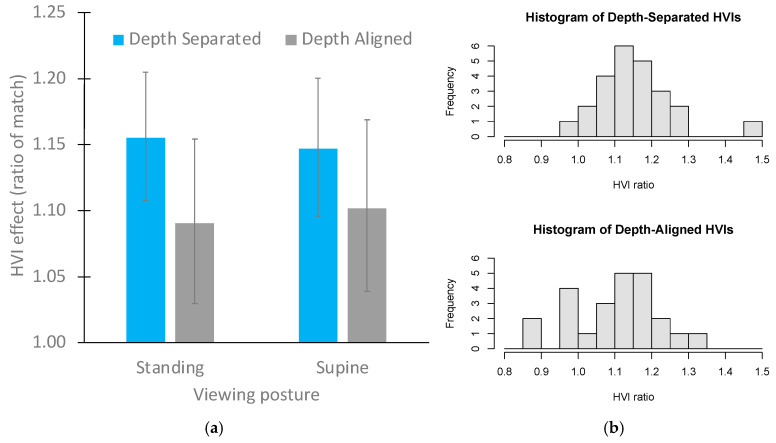
(**a**) The horizontal–vertical illusion as a function of depth separation and posture in Experiment 1. Error bars represent 95% confidence intervals. (**b**) Histograms show that the distribution of HVI ratios is approximately normal for the depth-separated lengths, but potentially bimodal for the depth-aligned matchers, suggesting a mixture of strategies when the horizontal and vertical extents were at the same egocentric distance.

The third preregistered analysis called for using ANOVA to test for differences between supine and upright conditions (after first establishing that there were no order effects, which there were not). The mean HVIs are shown for each depth-separation condition in Figure 4a as a function of posture and depth alignment. HVIs did not differ as a function of viewing posture, with *F*(1, 46) = 0.01, *p* = 0.91, and η_g_^2^ = 0.001.

Exploratory Figure 4b shows that the distribution of HVI ratios was fairly normal (symmetrical) when the objects to be compared were physically separated in depth, but it shows evidence of bimodality when the two extents were presented at the same depth. This complex distribution is consistent with the idea that even when the extents were presented in a single depth plane, the size-at-a-distance comparison was used by many participants. However it suggests that at an alternative comparison strategy was used by some participants, that was only available when the two extents were not separated in depth.

### 3.2. Exploratory Analyses of the Large-Scale HVI Data

**Figure 5:**
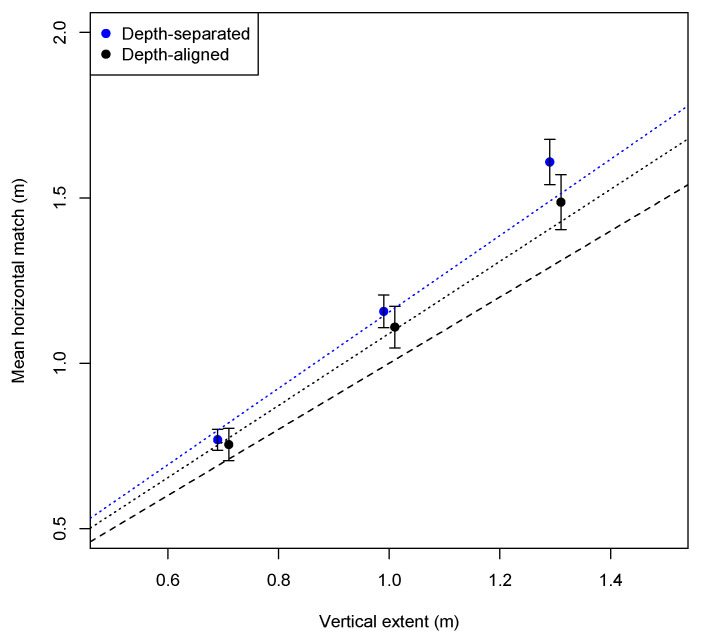
Grand mean horizontal matches of Experiment 1 as a function of pole height (linear-space analysis) in the two depth alignment conditions. Confidence intervals are shown based on the mean adjusted match for each of the 24 participants in each condition (collapsed across posture). The plot points are offset slightly left and right to avoid overlap. The dashed line represents an objective match. The dotted lines (colored according to the depth-alignment condition) have a zero intercept and are sloped with the mean HVI computed in the preregistered analysis in which the logs of the ratios pole were averaged.

The preregistration did not include predictions about the effects of pole height. However, as shown in Figure 5, HVI effects were strongest for the tallest (1.3 m) poles. In the depth-separated condition, the mean horizontal match for this pole height (analyzed in linear space) was 1.61 m, which represents a 1.23 HVI, and is reliably greater than the 1.5 m that would indicate a 1.15 illusion, with *t*(23) = 3.45, *p* = 0.002, and *d* = 0.56. Thus, for these tallest poles (near eye height), with the matcher separated in depth, the magnitude of the effect was essentially a 1.25 illusion, which is similar to the largest illusions reported for much larger outdoor objects [16,21].

### 3.3. Preregistered Analyses of Azimuth Estimates from Experiment 1

The final preregistered analysis for Experiment 1 concerned the estimates of azimuthal direction. A *t*-test conducted in log space (to make ratios additive) showed that azimuthal angle estimates had a much higher ratio to the actual angle for supine participants (M = 1.45) than for standing participants (M = 1.13), with *t*(44.4) = 3.61, *p* < 0.001, and *Cohen’s D* = 1.04. The expected value for azimuthal angles is about 1.2 [23]. The supine estimates reflect a ratio that is reliably greater than 1.25, with *t*(23) = 3.43, *p* = 0.002, and *d* = 0.70, whereas the standing estimates are not reliably different than 1.2, with *t*(23) = 1.1, *p* = 0.29, and *d* = 0.22. Despite there being no effect of posture on the HVI_LS_, the present results replicate the prior finding that explicit judgments of azimuthal angular direction are exaggerated with a gain of about 1.5 for supine observers [35], despite the fact that the present study used a real outdoor scene in which participants were able to make typical large-scale HVI matches.

Previous work on explicit angular estimates has suggested that they can be contaminated by other spatial information [39]. That is, despite the importance of angular information in the perception of space, and despite convergence between explicit and implicit measures of angular gain in standardized outdoor studies [23], explicit reports of angular direction do not always perfectly reflect the underlying angular variables that seem to control perception and action in space [39].

### 3.4. Preregistered Analyses of Azimuth Estimates from Experiment 2

The preregistered analysis path for Experiment 2 was to perform two initial ANOVAs to separately check for range effects and order effects while testing for effects of posture. Both these analyses confirmed that neither range nor order of testing were reliable factors (and both indicated that estimation slopes were higher when participants were supine). We followed these preliminary tests with the preregistered linear-mixed-effects regression (LMER) to estimate the effect of posture with range taken into account. LMER was chosen because it accounts for within-subject repeated measures while providing an overall slope estimate that can represent the gain that is not provided by ANOVAs.

The model showed that the mean slope in the standing condition was 1.06, whereas the slope of the estimates when supine was 1.26, which was significantly higher than the standing slope, with *t*(22) = 2.27, *p* = 0.04, and *d* = 0.47.

Although these slope values are both numerically lower than the angular gains found in Experiment 1, they replicate the observation that explicit azimuthal angle judgments of the same scene were more exaggerated when observers were supine than when they were standing. However, it remains possible that these angular estimate effects were affected by two or three distinct uncontrolled factors. First, the mirror used in Experiment 1 had a much larger aspect ratio in its field of view (FOV) than the mirror used in Experiment 2, and this may have contributed to producing the greater magnitude of the overestimation of azimuth angles in Experiment 1. Second, the range of angles used in Experiment 2 was larger (48° vs. ~35°), and this also could have contributed to producing a smaller gain overall. Third, and finally, even in the upright conditions, where the mirror was not used, the portion of the field being used in these experiments was bounded to the left and right by two trees that encroached upon the largest angles in Experiment 2 (see Figure 3b, for example, where the leaves of the trees on both sides are visible), but not in Experiment 1. Judgments of the azimuth angles may have been affected by these framing, bounding, and range effects in complex ways that may not have been as relevant to the HVI task. For example, the largest physical azimuthal angle involved in the HVI task (i.e., for the outermost anchoring point for matching the tallest pole at the nearest distance) would not have reached 30°, and nearly all frontal matches involved eccentricities less than 22° in azimuth.

The possible role of the aspect ratio of the effective FOV in explaining the classic HVI as well as in contributing to the overestimation of explicitly judged azimuth angles will be further considered in the Discussion.

## 4. Discussion

In the present study, we have successfully replicated an observation first reported in virtual reality [33], where presenting vertical and horizontal extents separated in depth produces large-scale HVIs with a gain of up to nearly 1.25. Such large HVI magnitudes have usually only been reported for very tall objects like buildings and light poles [16]. These effects seem to be consistent with the activation of processes for computing size at a distance rather than merely comparing projected size. In addition to showing that these effects occur in the real world, we have also successfully dissociated them from connectedness effects [6]. That is, whereas the previous report had used connected extents when testing at the same visual depth, we used disconnected extents in both conditions. Thus, Experiment 1 provides a somewhat clearer demonstration of the effects of separation in depth on the HVI than previous studies have.

It is well known that comparisons of egocentric ground distance to vertical extents show that vertical extents appear much larger than ground distances [24,25] and that horizontal frontal extents generally appear somewhat larger than egocentric ground distances [18,23,32]. One theory for this outcome is that perceived depth is foreshortened [32] and that vertical extents are exaggerated [19]. Some authors have sought to measure the HVI by separately measuring these comparisons between perceived height and perceived egocentric distance on the one hand, and between perceived width (frontal horizontal extent) and perceived egocentric distance on the other [17,18]. An alternative view is that biases in the perception of underlying angular variables (in elevation and azimuth) cause distortions in perceived egocentric ground distance. In particular, exaggerations in perceived elevation [22] clearly affect perceived angular declination. This is a well-documented source of information about ground distance [26,27,28]. Many studies show that the perceived 45° elevation direction relative to straight ahead is about 30° [21,22,24], implying a gain of 1.5 in perceived elevation. Similarly, the mismatch of frontal horizontal extents (matched to egocentric distances) can also be expressed in terms of angular distortions in the perceived azimuthal direction (e.g., implying that a perceived 45° direction corresponds to an actual azimuthal deviation of only 38° or a gain of 1.2) [17,23]. Thus, the size-at-a-distance strategy may involve comparing two frontal extents computed using angular variables that are differentially expanded in a ratio of 1:5:1.2, with a resulting bias of about 1.25.

### 4.1. Possible Effects of Field of View (FOV) Aspect Ratio on Explicit Estimation of Azimuthal Angles

The primary reason for choosing to test supine observers in both the HVI task and the azimuthal angle estimation task was prior evidence that the azimuthal direction was more exaggerated than normal for supine observers [35]. Specifically, the prior report found an angular gain of 1.5 instead of the more typical 1.2 or 1.25. Angular exaggeration in the azimuthal direction was found to be greater for supine observers than for upright observers in both Experiment 1 and Experiment 2. However, the observed gain was closer to 1.2 than to 1.5 in Experiment 2. A previous study suggested that there is a significant difference between explicit estimates of angular deviation and implicit measures of them [39], suggesting that explicit reports of direction can be affected by a variety of experimental conditions. Similarly, in the current study, there seems to be a dissociation between the effects of supine viewing on the HVI (an implicit measure) and on explicit measures of perceived azimuthal direction, and this may reflect something about factors that affect the explicit azimuth measure only.

Although our participants in both Experiment 1 and Experiment 2 showed greater overestimation of azimuthal angles when supine than when upright, these explicit angular errors did not translate into a change in the apparent matches between vertical and horizontal extents. (It is, of course possible, that they would also have more dramatically overestimated elevation angles as well, but this was not observed in an earlier report that first observed the effect of supine posture on judgments of the azimuth direction.) It thus seems likely that there is a dissociation between reported angular estimates and underlying perceptual gains that are affecting perceived size at a distance.

The previous report of azimuth exaggerations when supine speculated that it might be because egocentric azimuth when supine is also an elevation angle [35] relative to gravity. We might also speculate that establishing a reference frame for judging egocentric directions when supine may be somewhat disorienting because of the use of mirrors [40,41]. In particular, however, although the physical rectangular mirror used in the prior study and in Experiment 1 of the current study had an aspect ratio of nearly 3:1, which is already quite large, the additional compression of the shorter aspect of the field of view (since the mirror was at a 45° angle to the line of sight) meant that the effective aspect ratio of the rectangular mirror FOV was about 3.8 in Experiment 1 and in the prior report. A wide field of view was seen as an advantage in our original plan, but the abnormally large aspect ratio may have been the source of the larger-than-normal explicit azimuth estimates.

Both the aspect ratio of the framing stimulus and the aspect ratio of the FOV have been shown to affect the classic (2D) HVI [3,15]. With both eyes open, the binocular azimuthal field of view is greater than 180° (~214°) [42,43] whereas the field of view in elevation is much more limited (~125°), such that the aspect ratio of the normal binocular FOV is about 1.7. Thus, the aspect ratio of the FOV of the mirror used in the supine posture for Experiment 1 (3.8) was much larger than normal, and it was in this case that the azimuthal gain was found to be nearly 1.5. In Experiment 2, where a circular mirror was used, the effective FOV aspect ratio was about 1.4, which is much more similar to normal. In Experiment 2, the azimuth was found to have a gain of 1.27 for supine viewers using the mirror, which is more similar to that reported for observers standing upright in an open field (~1.2) [23].

Though the design of the experiments focused on the difference between supine and standing, there is clear evidence consistent with the effect of the FOV aspect ratio on the produced angular gains in the supine conditions of the two experiments. We sought to control for FOV in Experiment 1 by having people view the scene through the frame of the mirror when standing (see Figure 2a, above) as well as when supine. However, for standing observers, the visual scene outside the frame was visibly continuous with the scene within the frame, but this was not the case for supine observers. Thus, it appears that the prior report of the effects of being supine on azimuthal angles [35] may have been an artifact of the extreme aspect ratio of the viewing frame (which was also 3.8). Even so, it is, in some sense, all the more surprising that the measured HVIs were identical for supine and upright observers in Experiment 1, given that aspect ratio is known to affect the classic 2D HVI.

However, most studies of the HVI that have shown significant effects of the FOV have been conducted on the small-scale HVI [10,15], and at least one study has reported a dissociation between the effects on the (small-scale) HVI of aspect ratios (FOV) and of adding depth cues to the display [14]. Thus, the effects of aspect ratios on perceived azimuthal direction might be independent of the comparison of size at a distance. Similarly, it has been shown that a small-scale HVI is retinotopic. That is, the HVI anisotropy sticks with the orientation of the head [10]. In contrast, only a small part of the large-scale HVI rotates with the head; most of the large-scale effect occurs relative to the ground plane even for observers lying on their side while viewing the scene at a normal eye height [21].

If we assume that changes in FOV were what produced the differences in explicit azimuthal estimates, it seems reasonable to conclude that the large-scale HVI under investigation here was not affected by FOV as much.

### 4.2. Can Image Statistics Explain the Large-Scale HVI?

Similarly, the important idea that the classic HVI may be traced to biases in (2D) image statistics [12] has been applied experimentally only to small-scale (and 2D) HVI conditions. It is not clear how such a theory can explain the effect of merely separating the extents in depth, unless by removing a less-biased mode from what is normally a combination of multiple competing biases. Such a view would be consistent with other proposals that there are multiple processes involved in the large-scale HVI [14,21] rather than a single explanation. We note that in one prior study using a virtual environment [20], the same proximal visual information produced different magnitudes of HVI, depending on the perceived scale of the scene being viewed; this was manipulated in the virtual environment by either approaching a simulated monitor or a simulated outdoor scene. This latter finding is, again, interpretable in terms of a task strategy involving comparing perceived size at a distance vs. a task comparing relative projected size. This finding, like the current one, emphasizes the multiplicity of processes that may be responsible for the HVI.

### 4.3. Implications for Studying the Large-Scale HVI Using fMRI

The use of supine observers is often required in fMRI contexts. The present study used fields of view that were larger than 90° in azimuth but had a large aspect ratio. Such fields of view should be possible in fMRI situations using lens technology commonly used for head-mounted VR as well as specialized mirror/projection systems. Our observations were that the large-scale HVI did not differ between supine and upright observers when using these limited fields of view, but that other measures (such as explicit angular direction estimates in azimuth) were likely affected by the aspect ratio of the FOV.

A previous study with participants viewing the world with a mirror apparatus similar to ours reported an altered sense of body orientation—as if leaning slightly upward while viewing an “upright” world [44]. However, their participants (1) were not allowed to view the apparatus that they were placed in and (2) intentionally had pressure applied to the soles of their feet by a “foot board”, simulating a gravitational force along the long axis of their body (which, alone, may account for the illusory body orientation effect). We did not ask our participants to report their perceived body orientation, but no one reported any sense of tilting up at an angle when lying flat on our apparatus, nor did we notice such an effect when testing the apparatus ourselves.

### 4.4. Conclusions

What can be concluded from the present studies then is that comparisons of vertical and horizontal extents in locomotor space (on a ground plane and viewed from a normal eye height) seem to differentiate between biased processes that are based on projected (2D) size and those that are based on perceived size at a distance. In the case of lines drawn in a frontal plane, the magnitude of the horizontal–vertical illusion is typically about 1.1, whereas with real objects evaluated in spaces such as those that afford locomotion, the magnitude of the effect can be as much as 1.25 for objects that are no taller than a person. Most importantly, it appears that participants can be forced to take a more biased perspective simply by separating the objects in depth so that direct comparison of projected size becomes ineffective. It would be interesting to know if this would work with much smaller objects presented on a table surface, for example.

## Data Availability

Full data and analysis files are available on OSF (https://osf.io/zgjv3/?view_only=2d1bf1b2e9f947cca84c1a0fe4fe20cd).
